# Plasmodium copy number variation scan: gene copy numbers evaluation in haploid genomes

**DOI:** 10.1186/s12936-016-1258-x

**Published:** 2016-04-12

**Authors:** Johann Beghain, Anne-Claire Langlois, Eric Legrand, Laura Grange, Nimol Khim, Benoit Witkowski, Valentine Duru, Laurence Ma, Christiane Bouchier, Didier Ménard, Richard E. Paul, Frédéric Ariey

**Affiliations:** Institut Pasteur, Génome et Génomique des Insectes Vecteurs, Paris, France; Institut Pasteur du Cambodge, Epidémiologie Moléculaire du Paludisme, Phnom Penh, Cambodia; Institut Pasteur, Génétique Fonctionnelle des Maladies Infectieuses, Paris, France; Institut Pasteur, Plate Forme Génomique, Paris, France; INSERM U 1016, Institut Cochin, Université Paris Descartes Sorbonne Paris Cité, Faculté de Médecine, Paris, France

**Keywords:** Malaria, Anti-malarial drug resistance, Copy number variation, Bioinformatics

## Abstract

**Background:**

In eukaryotic genomes, deletion or amplification rates have been estimated to be a thousand more frequent than single nucleotide variation. In *Plasmodium falciparum,* relatively few transcription factors have been identified, and the regulation of transcription is seemingly largely influenced by gene amplification events. Thus copy number variation (CNV) is a major mechanism enabling parasite genomes to adapt to new environmental changes.

**Methods:**

Currently, the detection of CNVs is based on quantitative PCR (qPCR), which is significantly limited by the relatively small number of genes that can be analysed at any one time. Technological advances that facilitate whole-genome sequencing, such as next generation sequencing (NGS) enable deeper analyses of the genomic variation to be performed. Because the characteristics of *Plasmodium* CNVs need special consideration in algorithms and strategies for which classical CNV detection programs are not suited a dedicated algorithm to detect CNVs across the entire exome of *P. falciparum* was developed. This algorithm is based on a custom read depth strategy through NGS data and called PlasmoCNVScan.

**Results:**

The analysis of CNV identification on three genes known to have different levels of amplification and which are located either in the nuclear, apicoplast or mitochondrial genomes is presented. The results are correlated with the qPCR experiments, usually used for identification of locus specific amplification/deletion.

**Conclusions:**

This tool will facilitate the study of *P. falciparum* genomic adaptation in response to ecological changes: drug pressure, decreased transmission, reduction of the parasite population size (transition to pre-elimination endemic area).

## Background

The burden of malaria has decreased by half over the last decade. This is a direct consequence of effectives strategies mainly focused on vector control (long-lasting impregnated bed nets) and the management of suspect malaria cases (early diagnosis by rapid diagnostic tests and effective and prompt treatment with artemisinin-based combination therapy). As a consequence, a drastic decrease in *Plasmodium falciparum* population biomass in many countries has been observed [1]. This new epidemiological situation has led to a change in the environment within which the parasite finds itself and will thus alter the selective pressures on parasite populations.

Natural evolution of malaria parasites generates an enormous amount of genetic diversity either linked with copy number variations (CNVs), or acquisition of new single nucleotide variations (SNVs) and their accumulation over time [2]. This allows parasites to acquire a high capacity of adaptation to the environmental shifts and develop anti-malarial drug resistance. Indeed, SNVs are known to be at the origin of resistance to anti-malarial drugs, such as chloroquine, sulfadoxine, pyrimethamine, atovaquone, artemisinin, and *mdr1* gene amplification is known to be at the origin of mefloquine resistance [3–5].

In eukaryotic genomes, SNP mutation rates occur at a rate of ~10^−8^ per generation and deletion or amplification rates have been estimated to be in the order of ~10^−4^ per generation [6, 7]. The number of *P. falciparum* parasites infecting an adult can be estimated from 5 to 50 billion parasites (10^3^–10^4^ parasites per μL of blood with a total of 5 l of blood). Because asexual replication occurs every 48 h, the erythrocytic stage of *P. falciparum*, therefore, appears to be a breeding ground for any selection pressure to act on parasite population. Although the regulation of gene expression in *P. falciparum* is still incompletely understood, relatively few transcription factors have been identified [8, 9] and the regulation of transcription is seemingly largely influenced by gene amplification events. Thus CNV is a major mechanism enabling parasite genomes to adapt to new environmental changes.

Currently, the detection of CNVs is based on quantitative PCR (qPCR), which is significantly limited by the relatively small number of genes that can be analyzed at any one time, and the fact that endogenous controls (e.g., housekeeping genes) can introduce bias into the results if not properly chosen [10]. Technological advances that facilitate whole-genome sequencing such as Next Generation Sequencing (NGS) enable deeper analyses of the genomic variation to be performed. Because the characteristics of *Plasmodium* CNVs need special consideration in algorithms and strategies for which classical CNV detection programs are not suited, a dedicated algorithm to detect CNVs across the entire exome of *P. falciparum* based on a custom read depth strategy through NGS data was developed. This algorithm was named PlasmoCNVScan.

This study analysed CNV on three genes known to have different level of amplification and which are located either in the nuclear, apicoplast or mitochondrial genomes. The results showed a correlation between PlasmoCNVscan and the qPCR experiments, usually used for identification of locus specific amplification/deletion. The use of such a tool for the exploration of adaptive phenomena based on whole genome data is then discussed.

## Methods

### DNA

Real time PCR and whole genome analysis were carried out on the same DNA extracted from samples of *P. falciparum* collected in Cambodia between 2010 and 2014 and adapted to culture. DNA extraction was performed using QIAamp DNA Blood Kit (Qiagen ©).

### qPCR

The protocol for qPCR copy number evaluation used in this study was based on the WWARN (MOL-05) procedure: “Copy number estimation of *P. falciparumpfmdr1* v1.1''. Relative quantification was performed by using “PCR Applied Biosystem ViiA 7^®^” and the Taqman^®^ technologies (Thermo Fisher©).

An evaluation of the *pfmdr1, clcp* (PFC10_API0060) and *cytochrome b* genes was performed because they are all known to have CNV and belong to the three genomes (respectively from nuclear, apicoplast and mitochondrial genomes). The reference gene selected was the nuclear beta-tubulin-encoding gene (PF10_0084). The primers and probe used for the qPCR are described in the Table 1.

**Table 1:** The primers and probe used for copy number quantification

Name	Sequence	Gene amplification	Location
CytbF	5′GCACGCAACAGGTGCTTCTC 3′	*Cytochrome* b	Mitochondira
CytbR	5′GACCCCATGGTAAGACATAACC 3′		
CytBP	5′(FAM)-CCATGATAATGGTAAATACATATATGAGTAATTT-(TAMRA) 3′		
CLCPF	5′GGGCCTAGTGGTACTGGTAA 3′	*clcp*	Apicoplast
CLCPR	5′CCAACATAACCAGGAGGTGAACC 3′		
CLCPP	5′(FAM)-CATATCAAATCTAATTAGTTCTTTTTCAGAACC-(TAMRA) 3′		
Mdr1F	5′ TGCATCTATAAAACGATCAGACAAA 3′	*pfmdr1*	Nuclear
Mdr1R	5′ TCGTGTGTTCCATGTGACTGT 3′		
Mdr1P	5′ (FAM)-TTTAATAACCCTGATCGAAATGGAACCTTTG-(TAMRA) 3′		
TubF	5′AAAAATATGATGTGCGCAAGTGA 3′	*Pftubulin*	
TubR	5′AACTTCCTTTGTGGACATTCTTCCT 3′		
TubP	5′ (TET)-TAGCACATGCCGTTAAATATCTTCCATGTCT-(TAMRA) 3′		

All samples were analysed in triplicate. The confidence intervals on measures must be superior to 95 % for one triplicate and the Z-score, designating the deviation from a normal distribution, must be inferior to 1.75 (Life Technologies Corporation, 2011). All the samples results that did not meet these criteria were removed from the final results.

### Whole genome

Whole-genome sequencing was performed on parasite DNA from Cambodian parasite isolates, using an Illumina paired-reads sequencing technology, as previously described [11].

### PlasmoCNVScan

Read depth-based methods have recently become a major approach for estimating copy number [12]. The underlying concept of RD-based methods is that the depth of coverage in a genomic region is correlated with the copy number of the region; e.g., a lower than expected depth of coverage intensity indicates deletion and a higher than expected depth of coverage intensity indicates amplification [13]. The algorithm in classical RD-based methods relies heavily on the assumption that the sequencing process is uniform, i.e., the number of reads mapped to a region is assumed to follow a Poisson distribution and is proportional to the number of copies [12]. However, due to the GC content and “mapability”, this assumption is for the most part unrealistic. Moreover, the uneven representation of genomic regions in library preparation due to variability in DNA fragmentation may induce a bias [14].

**Fig. 1:**
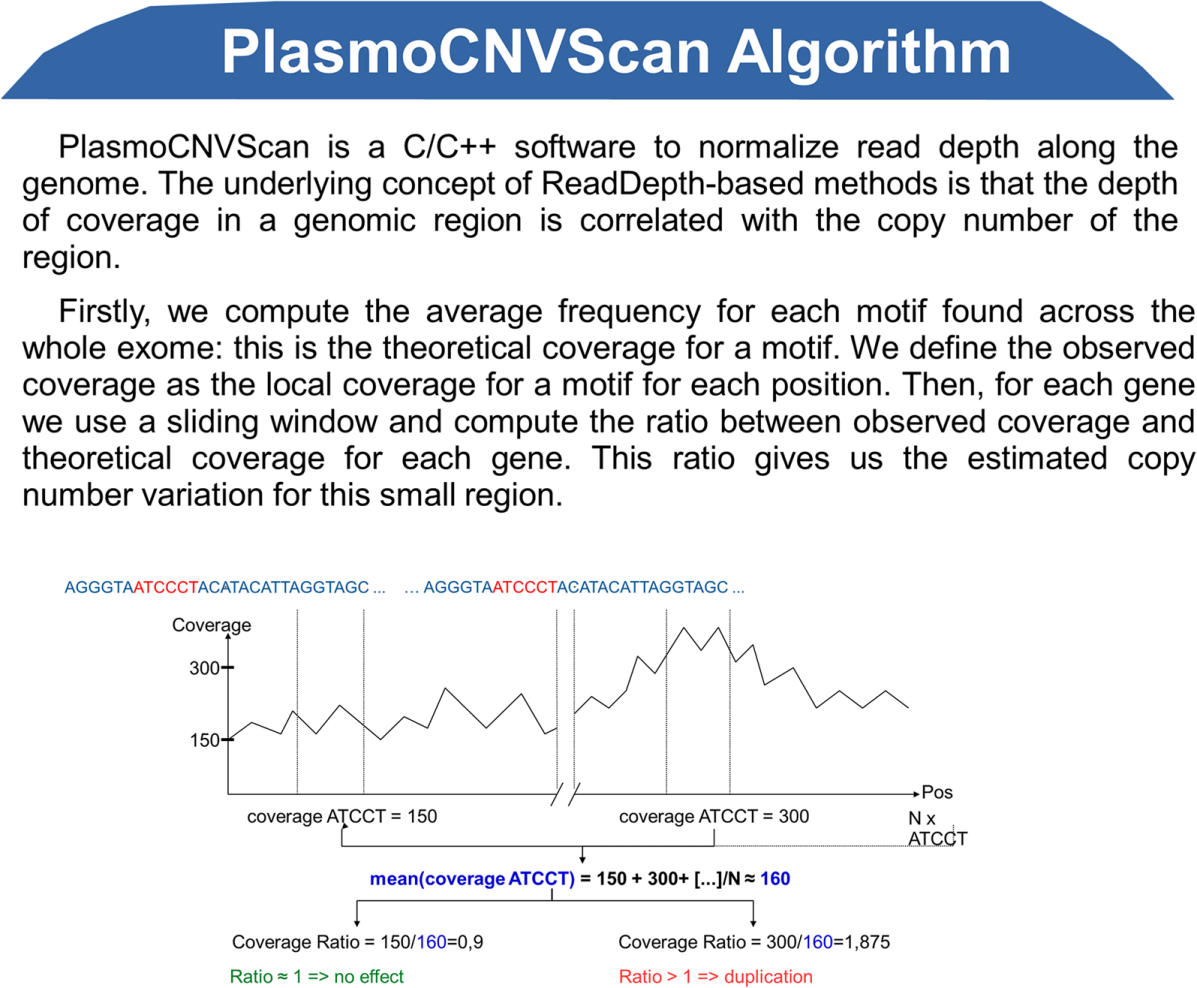
PlasmoCNVScan algorithm

In PlasmoCNVScan this assumption is by-passed using sequence pattern coverage across the overall exome. The reads must be correctly mapped onto a well-annotated reference genome. The main hypothesis is that the depth of coverage for each motif in the exome only depends on the sequence and thus has the same intensity. Here, a motif represents a subset of a fixed number of nucleotides in the genome. The motif’s coverage is the average coverage of this subset (see Fig. 1).

Firstly, the average frequency for each motif found across the whole exome was computed: this is the theoretical coverage for a motif. The observed coverage is the local coverage for a motif for each position (extracted from pileup file). Then, for each gene, using a sliding window, the ratio between observed coverage and theoretical coverage for each gene/position was computed. This ratio gives the estimated copy number variation for this region.

The algorithm was implemented in homemade software in C language called PlasmoCNVScan. PlasmoCNVScan use the external libraries gbfp [15] under GNU GPL v2 licence and utash.under the revised BSD licence.

### Optimising the size of the sequence length used for the motif

**Fig. 2:**
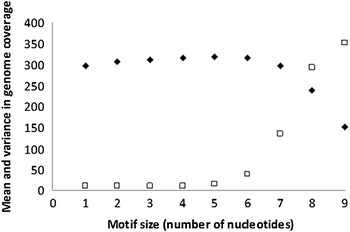
Motif size, mean and variance relation. Mean coverage of the genome is represented as *filled squares* and variance coverage of the genome is represented as *open squares*. Variance is divided by 100 for clarity

The length of the motif is arbitrary, but clearly a motif of size 1 nucleotide will completely cover the genome but will yield no information on intra-genomic variation, whereas a size of hundred nucleotides will lead to little coverage and huge variation in the coverage across the genome. The motif size was increased from 1 until the variance in the coverage among intra-genomic region increased. Using the reference genome, *P. falciparum* clone 3D7, the optimal number of nucleotides for the motif was assessed. As can clearly be seen in Fig. 2, the variance increases significantly after a motif length of 6 nucleotides. The optimal motif size appears to be 5 or 6 nucleotides. The size of the motif was set to 6.

### PlasmoCNVScan versus benchmark softwares

The dataset was tested for *pfmdr1* gene with two programs for detecting copy number variation using next generation sequencing data. CNV-seq [16], which is widely used software in case–control studies, and CNVnator [17], which uses a similar approach to calculate RD signal and correct the GC-bias.

### Statistical analysis

The qPCR results were considered as reference and the Pearson test was used to calculate the measure of the linear correlation (dependence) between the two variables qPCR and PlasmoCNVScan or CNVnator software, giving a value between +1 and −1 inclusive, where 1 is total positive correlation.

## Results

**Fig. 3:**
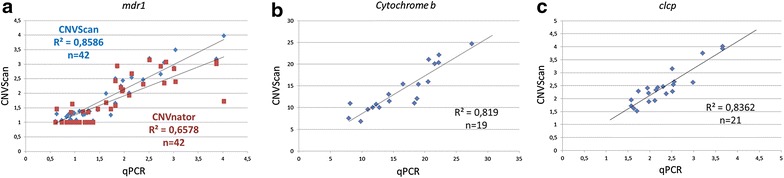
Correation coefficient between qPCR and PlasmoCNVScan results for *mdr1* (**a**), *cytochrome b* (**b**) and *clcp* (**c**). Correlation coefficient between qPCR and CNVnator was added in *red* for *mdr1* (**a**)

According to the results of the copy number obtained for 19 isolates (*cytochrome b* gene, mitochondrial genome), 21 isolates (*clcp* gene, apicoplast genome) and 42 isolates (*pfmdr1* gene, nuclear genome) with real-time PCR, a correlation line was established with the results from PlasmoCNVScan tool on illumina FASTq files. As can be seen in Fig. 3, R^2^ values for the two types of extra nuclear genome and for nuclear genome are greater than 0.8. Moreover, the equation obtained type y = ax + b has a factor “a” close to 1 with a very low b value, tending towards the type y = x; thus both methods are proportional to each other and tend to be similar.

### PlasmoCNVScan vs CNV-seq and CNVnator

#### CNV-seq

As CNV-seq method is conceptually derived from array comparative genomic hybridization (aCGH), two sets of reads mapped onto the same reference genome from the same flow cell is needed. CNV-seq fails to detect CNV on all isolates, because 3D7, used as a reference, has been sequenced on a different flow cell to the other isolates. To avoid this problem, there is a need to include a reference isolate in each of the flow cells used, which becomes prohibitively expensive.

#### CNVnator

CNVnator is able to discover CNVs in a vast range of sizes, from a few hundred bases to megabases in length for a single genome. The correction of GC-bias is based under the observation that the RD signal and GC content are correlated. Strikingly, CNVnator had a lower correlation with qPCR than PlasmoCNVScan (R^2^ = 0.65, N = 42 Fig. 3).

## Discussion

The overall (A + T) composition is 80.6 % in the *P. falciparum* genome and increases to ~90 % in introns and intergenic regions [18], resulting in very high similarity among non-coding regions. This introduces an important bias for CNV identification using NGS data. In coding regions, the GC content is higher and the coverage is likely to be higher and more specific. This heterogeneity in the GC content between coding and non-coding sequences led us to compute the average coverage for exons only.

For computing the CNV on a single sample using PlasmoCNVScan, only a BAM file (which is converted in pileup file) is necessary, along with the reference genome (fasta file) and a gff file. Given mapped reads, the efficient implementation of PlasmoCNVScan allowed a non IT specialist to perform whole-exome analysis of *P. falciparum* within a few minutes on a single 3.3-GHz Intel Core 3 Duo CPU. The RD signal is normalized with the genome itself. PlasmoCNVScan is thus able to compare different CNV exomes from different experiments. The results show very good correlation with the qPCR results, with R^2^ value above 0.8 for all the three genes explored irrespective of the CNV range (from 1 to 30 in the case of *cytochrome b* mitochondrial gene).

**Fig. 4:**
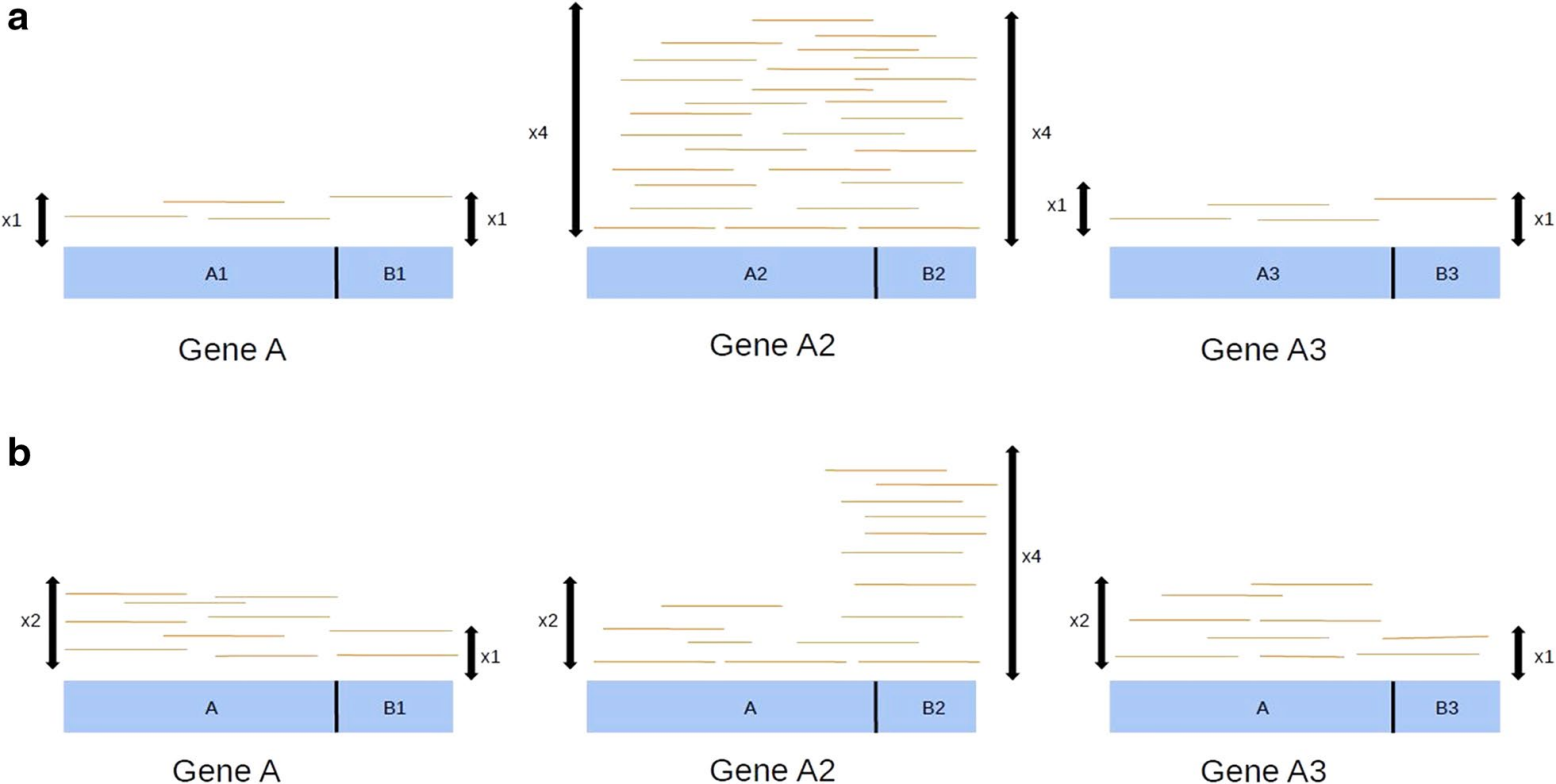
Expected coverage ratio (**a**) and observed coverage ratio (**b**) in a multigene family

The main limitation of the algorithm is that when facing multigene families biases could appear for gene amplification detection or for the precise identification of the gene really amplified. Figure 4 shows an example in the case of a multigene family. The three genes share a common sequence (A) and a variable sequence (B1, B2, B3). The reality is shown in Fig. 4a: genes A1 and A3 are not amplified, gene A2 harbors four copies, thus the ratio given by PlasmoCNVScan should be four. The observed computed ratio is shown in Fig. 4b. Because of the A common sequence, reads are equally distributed among the multigene family and the computed ratio is 2 : (4+1+1)/3 = 2. The computed ratio for specific regions (B1, B2, B3) are correct. In the case presented in this paper the *clcp*, *cytochrome b* and *pfmdr1* genes showed no significant common nucleotide sequences with other genes to scramble information. Confirmation by qPCR targeting specific areas of the studied genes would circumvent this problem.

However when working with polyclonal infections, which is a very common situation in Africa, the same problem may arise in the case of mixed infections with different parasites that do not possess the same CNV profile. In this case the qPCR will be of no help.

## Conclusions

The aim of this study was to test the ability of the algorithm to calculate the CNVs based on a whole genome sequencing with small reads (FASTQ). Thus the authors chose to work on clonal isolates directly isolated from the field (not reference strains). The Cambodian isolates were previously culture adapted (only for several cycles) before DNA extraction, likely leading to the removal of minor clones. The exome approach generates even more accurate data because of the higher GC content of the coding regions than in the intergenic regions, and, of course, expressed genes have much less similarity among them.

The strong correlation found between classical qPCR and PlasmoCNVScan opens the way for a systematic screening of CNVs changes on whole exomes. The global analysis of changes in the *P. falciparum* exome CNVs is beyond the scope of this article, but it is hoped that PlasmoCNVScan can be a useful tool to explore *P. falciparum* genomic adaptation in the face of ecological changes: drug pressure, decreased transmission, reduction of the parasite population size (transition to pre-elimination endemic area).
